# Rabies in Henan Province, China, 2010–2012

**DOI:** 10.3201/eid2002.131056

**Published:** 2014-02

**Authors:** Guo-Wei Li, Zhen-Yu Qu, Alfred King-Yin Lam, Jian-Guo Wang, Feng-Lan Gao, Tong-Xing Deng, Jia-Hai Lu

**Affiliations:** Zhengzhou Center for Disease Control and Prevention, Zhengzhou, China (G.-W. Li);; Luohe Medical College, Luohe, China (Z.-Y. Qu, J.-G. Wang, F.-L. Gao,T.-X. Deng);; School of Public Health, Sun Yat-sen University, Guangzhou, China (G.-W. Li, J.-H. Lu);; School of Medicine and Griffith Health Institute, Griffith University, Gold Coast, Queensland, Australia (A.K.-Y. Lam).

**Keywords:** rabies, rabies virus, viruses, zoonoses, China, Henan Province

**To the Editor**: Rabies is considered a reemerging zoonosis in China because many cases have been reported in recent years (*1*). The first case of rabies in Henan Province was reported in 1951. No more than 10 cases were reported per year during 1995–2001. However, beginning in 2002, the number of cases increased exponentially each year, and reached >100 in 2005 (*2*). To identify the epidemic characteristics of rabies in Henan Province, we examined the archived data of cases during 2010–2012. The surveillance data were collected by the Henan Center for Disease Control and Prevention (CDC) through systematic reporting and reports from sentinel hospitals.

Henan Province is situated in the mid-eastern region of China between northern latitudes 31°23′–36°22′ and eastern longitudes 110°21′–116°39′. The climate zone spans from warm temperate to subtropical, is humid to semi-humid with risk for monsoons, and has average annual temperatures ranging from 12°C to 16°C. The province occupies an area of 165,994 km^2^ divided into 18 municipalities, which are subdivided into 159 county-level divisions. Its population was reported to be ≈94 million in 2010 (*3*).

During 2010–2012, a total of 94 cases of rabies in humans were reported in Henan Province. Rabies was diagnosed in almost all of those cases in sentinel hospitals on the basis of clinical features of the disease. Rabies was confirmed in <10 patients by using a rapid fluorescent focus inhibition test in the Henan CDC. This test is performed by mixing different dilutions of test serum samples with a constant number of rabies virus isolates in a multichambered slide (*4*).

The number of patients in whom rabies was diagnosed in 2010, 2011, and 2012 were 31, 31, and 32, respectively. Thus, the prevalence of the disease during this period was 0.031 cases/100,000 inhabitants. There was no significant difference in occurrence of the disease during each year of the study period (χ^2^ = 0.021, p = 0.989). Rabies was reported in 13 (72.2%) of 18 municipalities and 53 (33.3%) of 159 counties (Technical Appendix  Figure).

Patients who tested positive for rabies were reported during all seasons, but more were reported during summer. Of the 94 cases, 33 (35%) were reported in summer months; the largest number of cases in 1 month (n = 15) was reported during September.

The age of the patients with rabies ranged from 2 to 83 years. The disease was most often found in persons 45–60 years of age (n = 36) and was reported least frequently in persons 15–30 years of age (n = 4). The male-to-female ratio of persons in whom rabies was diagnosed was 1.9:1.0 (n = 62 males, 32 females). The age and sex of these patients were similar to those of rabies-infected persons reported in the past in Henan or in other parts of China (*5**,**6*). Nearly all the patients with rabies were exposed to the virus through dog bites; 1 patient was exposed by being scratched by a cat. Most patients (n = 87 [92.5%]) died within 2 days of being exposed; 3 patients (3.2%) died within 9 days. The date of death was not recorded for 4 patients (4.3%).Three patients who died had received rabies vaccine after being bitten by dogs.

Rabies is a fatal infectious disease and remains a public health problem in China (*1*). In Henan Province, the increase in the number of rabies cases parallels the increase in the number of dogs (*7*). Nearly one third of China’s provinces reported rabies in this survey; the number of cases reported in the central region was much higher than that in other regions (Technical Appendix Figure). This may be related to the growing economy in the central region and a resulting increase in the number of people who keep dogs.

With regard to patient characteristics, the proportions of farmers, children, and students who had rabies were 72.3%, 9.6%, and 12.8%, respectively. The larger number of infected farmers could have been affected by many factors. The high cost of rabies vaccine and the lack of prompt treatment were related to many additional deaths. The number of children and student case-patients was 21 (22.4%). This high percentage may indicate that these groups need additional protection. Regarding the patient who was infected by cat scratch, there had been no reports of rabies in cats in China before this incident (*8*). Thus, we should be aware of this possible route of infection.

In summary, the severity of rabies and its increased incidence present a public health threat, and appropriate control strategies in Henan province are needed. A new rabies control system should be established that includes cooperation of the various health care sectors to provide protection to the public.

Technical Appendix Distribution of rabies cases among counties of Henan Province, China, 2010–2012.
