# Supplementary material for: Rabies in Henan Province, China, 2010–2012
Source: Emerg Infect Dis. 2014 Feb;20(2):331–3. doi: 10.3201/eid2002.131056 (PMC3901497; doi:10.3201/eid2002.131056)
Supplement: Technical Appendix — Distribution of rabies cases among counties of Henan Province, China, 2010–2012. [file 13-1056-Techapp-s1.pdf]

Article DOI: http://dx.doi.org/10.3201/eid2002.131056

Rabies in Henan Province, China, 2010–2012

Distribution of Rabies Cases among Counties of Henan Province, China, 2010–2012.

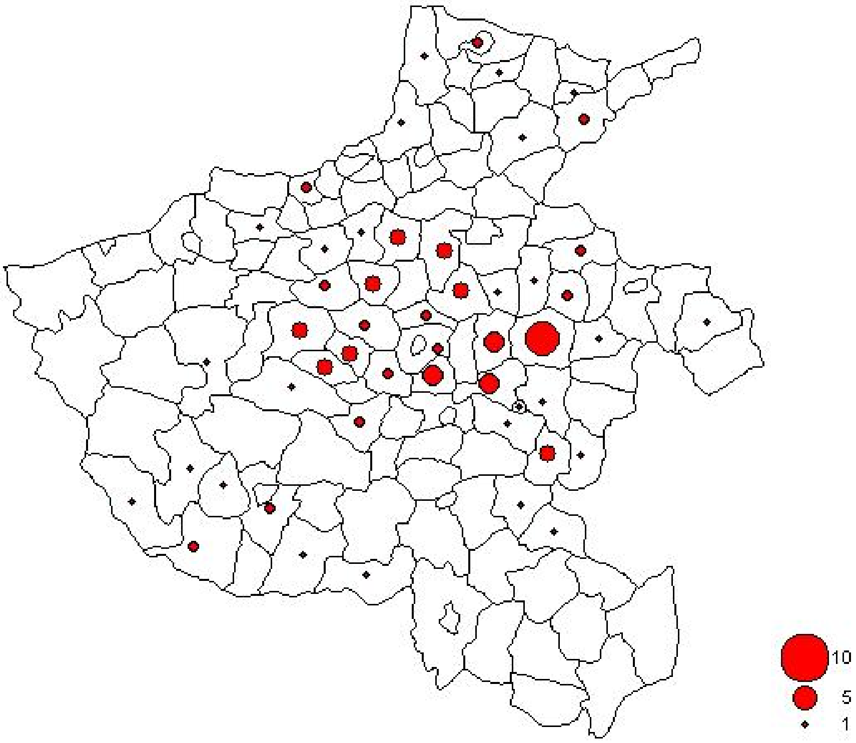

Figure. Rabies cases in counties of Henan Province, China, 2010–2012. Circles represent the numbers of rabies cases in each county during the study period.
